# The sexually driven epidemic in youths in China’s southwestern border region was caused by dynamic emerging multiple recombinant HIV-1 strains

**DOI:** 10.1038/srep11323

**Published:** 2015-07-02

**Authors:** Huamian Wei, Hui Xing, Jenny H. Hsi, Manhong Jia, Yi Feng, Song Duan, Cui He, Shitang Yao, Yuhua Ruan, Xiang He, Lingjie Liao, Yanling Ma, Yunda Huang, Lin Lu, Yiming Shao

**Affiliations:** 1 State Key Laboratory for Infectious Disease Prevention and Control, Collaborative Innovation Center for Diagnosis and Treatment of Infectious Diseases, National Center for AIDS/STD Control and Prevention, Chinese Center for Disease Control and Prevention, Beijing, China; 2 Yunnan Center for Disease Control and Prevention, Kunming, Yunnan, China; 3 Dehong Center for Disease Control and Prevention, Dehong, Yunnan, China; 4 Guangdong Provincial Institute of Public Health, Guangdong, China; 5 Fred Hutchinson Cancer Research Center, Seattle, USA

## Abstract

Dehong prefecture, Yunnan province on China’s southwestern border was the gateway of the country’s AIDS epidemic. Studies on HIV-1 molecular epidemiology will provide key information on virus transmission dynamics and help to inform HIV prevention strategies. HIV-1 infected youths (age 16–25 years) diagnosed in the continuous 3 months in 2009 to 2012 were enrolled. By means of phylogenetic and statistical analyses, It was showed that two thirds (133/205) of youths in Dehong, of which 74.1% were infected sexually, were infected by uncharacterized recombinant HIV-1 strains. Among them about 59.4% (79/131) were unique recombinant forms (URFs) and 40.6% (54/131) formed 11 transmission clusters, termed potential circulating recombinant forms (pCRFs). The emergence of recombinants was statistically significant related with people of low education, residents outside the capital city of Dehong and being Myanmar residents. It was the first report with ongoing HIV-1 recombinant strains in a sexually driven epidemic area in China. Great efforts should be put on reducing multiple risk exposures behavior in local young people, containing the spread of pCRFs to other regions, and preventing the URFs from evolving into future CRFs. Collaborative prevention across border is needed to better control the local AIDS epidemic.

HIV-1 remains a global threat to health and wellbeing, with an estimated 35.3 million people living with HIV in 2012 ( http://www.unaids.org/). Although the number of newly infected people has declined since 2001, viral diversity is increasing^1^. HIV diversity can be attributed to its high rate of mutation and virus replication as well as recombination between different viral strains. The identification of a circulating recombinant form (CRF) is defined through at least three epidemiologically unlinked individuals with full genome sequences displaying the same recombinant structure, while those that do not meet this criterion are categorized as unique recombinant forms (URFs)^2^. Currently, more than 65 CRFs have been included in the HIV Database ( http://www.hiv.lanl.gov), and an even greater number of URFs has been reported. The increasing complexity of local and global HIV epidemics brings challenges to effective diagnosis, antiretroviral treatment, disease progression monitoring, and virus transmission prevention^1^.

The HIV epidemic in China has become increasingly genetically complex, with subtypes B, B’, C, CRF0_1AE, CRF07_BC, CRF08_BC, and other strains co-circulating^3^. In particular, Dehong prefecture in Yunnan province, southwest China, is considered the gateway of China’s HIV-1 epidemic, from where most of the HIV-1 strains currently circulating in the country had first appeared^4^,^5^,^6^,^7^,^8^,^9^. The Dehong government has organized an impressive AIDS prevention campaign, and was the first prefecture in China that achieved a stable and then declining cases of HIV infections, a sign of the epidemic containment. Nevertheless, new recombinant strains are continually reported there among key high-risk populations such as injecting drug users (IDUs)^10^,^11^. HIV recombination is more commonplace in those infected through parenteral routes as compared with sexual routes, since the mucosal barrier in the latter poses a greater genetic barrier against dual and multiple infection^12^. Recombination in IDUs pose fewer concerns for generating a widespread recombinant epidemic in China, as their HIV-1 incidence has been found in decline in most regions of the country^13^,^14^. There is seldom report thus far on high proportions of recombinant HIV-1 found in sexual transmission networks, which has become the major driving force in China’s current AIDS epidemic. At the same time, existing reports on recombination also often lack appropriate sampling methods aiming at recent HIV infections, and hence may not accurately represent the current HIV epidemic.

This study design is based on the WHO Threshold Survey for transmitted HIV drug resistance surveillance that sought to capture recent infections by sampling from newly reported HIV infections in people less than 25 years of age. It aims to capture the full picture of the current ongoing epidemic in Dehong through characterizing the scale and patterns of recombinant HIV infections as well as factors related to their cause.

## Results

### Demographic characteristics of study population

The basic characteristics of the sample population were summarized in Table 1. Generally, the number of samples per year, the proportion of sex and the marital status were basically the same during study period of 2009–2012, as expected when an epidemic has reached plateau. Dehong is a multi-ethnic region with over half being non-Han ethnic minorities. Since cross-border migration regulations are relatively permissive, 17.6% of study subjects are Myanmar residents. Noteworthily, heterosexual transmission accounts for the vast majority of sampled subjects (74.1%), which is very similar to the heterosexually infected HIV positive population (74%, 4142/5608) reported during 2009–2012 in Dehong.

### Subtypes and recombinant patterns identification

**Figure 1:**
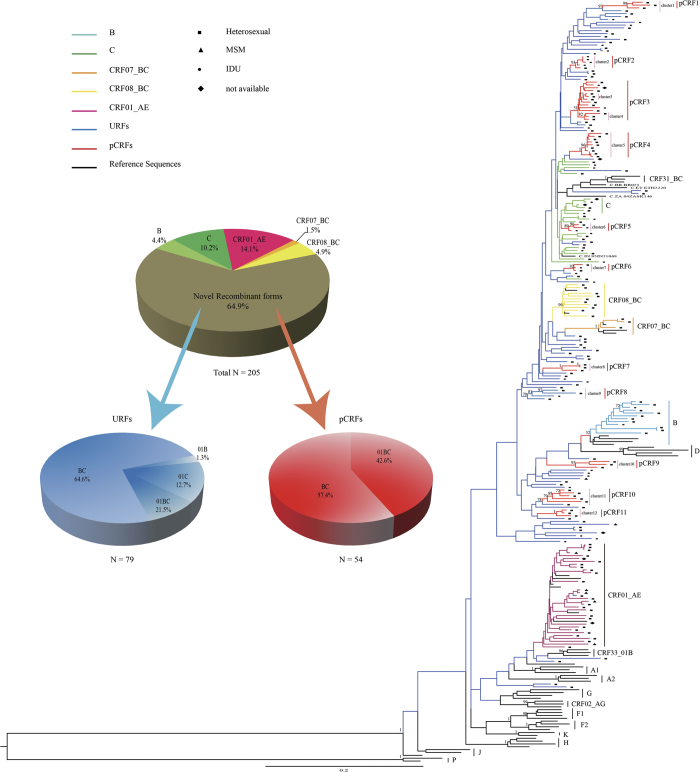
Phylogenetic analysis of the 1.2 kb *pol* gene fragments of 205 samples. Sequences were aligned and phylogenetic tree constructed using the maximum-likelihood method in PhyML. Strain designations are marked with different colors. Clusters with high bootstrap values and potential circulating recombinant clades (pCRFs, n = 11) are marked with vertical lines and labeled accordingly. Markers on the individual sequences indicate the route of transmission: ■, heterosexual sex; ▲, MSM sex; •, intravenous drug use; and ♦, not available. The proportion of each subtype is also shown in the figure.

**Figure 2:**
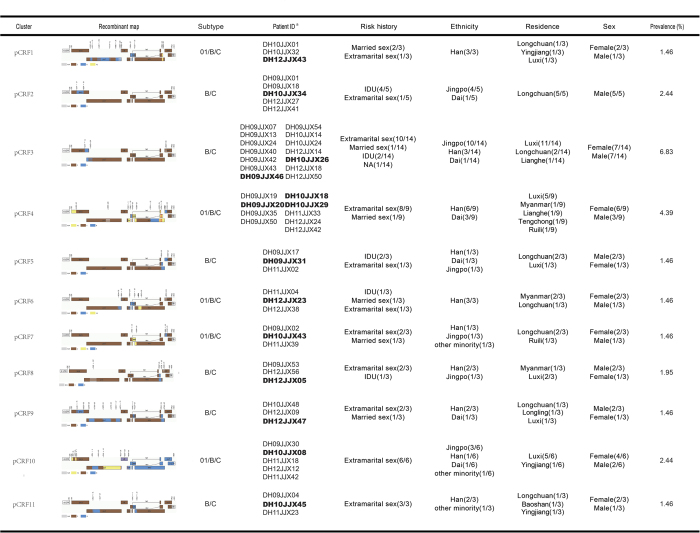
Characteristics of potential circulating recombinant forms. The recombinant patterns of the 11 pCRFs were listed, with brief summary of demographic information of each cluster. Patient IDs in bold indicate samples with NFLG sequences, and all other IDs indicate 1.2 kb *pol* gene fragments.

Phylogenetic analysis of 205 HIV-1 *pol* gene fragments showed that the majority of infections (64.9%) in 2009–2012 among young persons in Dehong prefecture consisted of not the conventional subtype group, but the new recombinant forms (Fig. 1, Table 2). This observation was confirmed by combined results of phylogenetic tree analyses, breakpoint analyses and near full-length genome (NFLG) (Fig. 2). The conventional subtypes account for 35.1% of all the samples, in which CRF01_AE was the most prevalent strain at 14.1%, followed by subtype C (10.2%), CRF08_BC (4.9%), B (4.4%) and CRF07_BC (1.5%) (Fig. 1). Among the new recombinant viruses, 12 clusters were identified from 54 subjects with at least three sequences in each cluster with bootstrap values no less than 0.7. The remaining new recombinants viruses from 79 subjects were interspersed widely throughout the phylogenetic tree. Bootscanning analysis was performed by both Simplot and RIP to identify recombinant breakpoints and confirm strain designations. The result was list in Supplemental Table S1. The new B’/C recombinants consisted 40% of the total sample, followed by CRF_01AE/B’/C (19.5%), CRF01_AE/C (4.9%), and CRF_01AE/B’ recombinants (0.5%) (Fig. 1).

### URFs and potential CRFs

For the 12 high-bootstrap clusters with non-conventional genotypes, NFLG sequencing was performed to clarify their genomic structures, obtaining a total of 15 NFLGs (Fig. 2), with at least one sequence in each cluster. One sequence at the root of cluster 11 was removed due to inconsistent structure with the rest of the cluster; while clusters 3 and 4 were found to have identical structures and merged at the outermost common branch point. Thus, these clusters with identical breakpoints were labeled as potential circulating recombinant forms (pCRFs) with consideration of the criteria for CRF. A total of 11 pCRFs consisting of 54 sequences (26.3% of the sample) were identified. The remaining 38.5% sequences (n = 79) were designated as URFs, since no phylogenic cluster can be formed among them. For pCRFs, only two recombinant patterns, 01/B’/C (n = 31) and B’/C (n = 23), were found. For URFs, all recombinant patterns observed in the study were found in the URFs, including B’/C (n = 51), 01/B’/C (n = 17), 01/C (n = 10) and 01/B’ (n = 1) (Fig. 1). Although recombinant structures vary widely, some sequences, especially B’/C recombinants, showed common breakpoints with each other despite being far apart on the phylogenetic tree; some URFs also shared breakpoints with CRF07_BC, CRF08_BC, or the pCRFs, suggesting common ancestry or related mechanisms of recombinant generation.

### Characteristics of potential CRFs

The structures and basic information on the pCRFs are summarized in Fig. 2. pCRFs1, 4, 6, 7, and 10 included fragments from subtypes B’, C, and CRF01_AE (n = 23/205, 11.2%), while the others contained only B’ and C fragments (n = 31/205, 15.1%). pCRF2 (n = 5) was highly associated with being male, IDU, Jingpo ethnicity, and permanent residence in Longchuan county within Dehong. The largest cluster, pCRF3, was strongly associated with permanent residence in Luxi county, the prefecture’s capital (n = 11/14), as well as being of Jingpo ethnicity and history of extramarital sex (n = 10/14). pCRF4 was found in those permanently residing in four Dehong counties and Myanmar, and was associated with extramarital sex (n = 8/9) and being female (n = 6/9), which may be related to commercial sex work. pCRF6 was exclusively found in those with Burmese permanent residence (n = 3), and pCRFs 1 and 6, both CRF01_AE/B’/C recombinants, were found exclusively in Han Chinese (n = 6). It is interesting that all pCRFs can be transmitted through heterosexual contact. The 01/B’/C recombinants were highly associated with sexual transmission (n = 22/23) as opposed to intravenous drug use. In contrast, nearly all B’C clusters in pCRFs were both circulating in IDU and heterosexual population.

The pCRFs sequences were blast for similarities to all known sequences in the Los Alamos HIV Sequence Database from Dehong, elsewhere in China, and the neighboring countries India, Myanmar, Thailand, and Vietnam. Phylogenetic analyses indicated few links between the current Dehong recombinant strains and historical transmissions elsewhere (Supplemental Figure S1). All pCRFs, except pCRF6, clustered closely with numerous sequences sampled from Dehong in 2005, 2009, and 2010^11^,^15^,^16^. pCRF6, which found solely in Burmese permanent residents, co-clustered only with sequences sampled from northern Myanmar in 2009^10^. In addition, pCRF4 also co-clustered with one sequence from Anhui province^17^, while pCRF9 with one sequence in Yunnan elsewhere^18^ and another one from neighboring Guangxi province^19^.

### Factors related with the generation of new HIV-1 recombinant strains

Chi-square tests between each subtype and the demographic risk factors were performed (Supplemental Table S2). To study the relationship between HIV subtype and demographic risk factors, both univariate and multivariate logistic regression analyses were performed (Table 2). Viral strains were grouped as conventional subtypes, pCRFs, and URFs, with consideration of the epidemic time and scope. The percentage of each category with particular factors is also summarized in Table 2. Univariate regression analysis showed that sampling year (2011: OR = 3.66, 95% CI = 1.37–9.84; 2012: 2.65, 1.05–6.68 ), ethnicity (Jingpo: 2.55, 1.14–5.71), education (primary school and no schooling: 3.39, 1.68–6.82), occupation (farmer: 2.32, 1.09–4.95) and residence (other Dehong county: 2.82, 1.30–6.11; Myanmar: 4.44, 1.78–11.1) were related with URFs category, which was mostly the same as chi-square analysis (Supplemental Table S3). Upon adjustment for multivariate logistic regression, only education (AOR = 2.41, 95% CI = 1.09–5.36) and residence (other Dehong county: 2.90, 1.28–6.60; Myanmar: 3.17, 1.20–8.39) remained significant factors. Generally, the URFs category which represents newly emerged recombined HIV strains was highly related with people with lower education levels and who were living in Myanmar or other reigns beyond the capital city of Dehong. However, no relationship was found between pCRFs category and factors refer to the conventional subtype’s category. And the distribution of new recombinant HIV-1 strains showed no significant differences among populations with different high-risk contact history.

## Discussion

Since the 1980s, Dehong prefecture in China’s southwest border near the drug producing “the Golden Triangle” has been a hotspot of HIV infection and recombination, with great impact on China’s overall HIV epidemic^5^,^6^. Numerous studies have described the prevalence of recombinant forms and their transmissions between neighboring regions^10^,^11^,^15^,^16^,^20^,^21^,^22^,^23^. However, these studies either constrained to a single group of population^10^,^11^,^20^ or failed to identify the current transmitting strains^20^,^21^ as well as explore the correlates of emerging recombinants^15^,^16^,^22^,^23^. Numerous studies reported high rates of recombinant HIV-1 found in IDUs^10^,^11^,^24^, since the parental strains with greater genetic similarity are more likely to recombine^25^. Infection caused by multiple founder viruses happened more often in IDUs compared heterosexually infected population^12^. Without the mucosal barrier in IDUs during transmission, it may also lead more easily to recombinant HIV-1 strains. There is rarely report for high proportions of recombinant HIV-1 strains in sexual driving epidemic in Asia. Two studies reported comparatively high proportions of recombinant HIV-1 strains in sexually driven epidemics in sub-Saharan Africa^26^,^27^, one with 25% found in a group of antiretroviral treated patients in Ghana, the other with 47% found in Democratic Republic of Congo(DRC) . However, these studies did not distinguish long term and recent infections, and thus the recombinants observed may not reflect the current epidemic.

In this study, it characterized the currently occurring epidemic of HIV-1 infections among young people in Dehong Prefecture in Yunnan Province of China. The sampling methodology was based on WHO’s threshold survey of HIV drug resistance strategy with strong supports by China’s public health infrastructure. All young people newly diagnosed in the first Quarter of each year by all county CDCs in Dehong Prefecture were enrolled into the study consecutively for 4 years, from 2009 to 2012. Our study found that two third (64%) of the newly diagnosed HIV-1 infections in Dehong was caused by new forms of recombinant HIV-1 strains. To our knowledge, this is reported for the first time very high proportion of new recombinant HIV-1 in predominantly heterosexually infected populations.

Among the newly described new recombinant forms, phylogenetic and breakpoint analyses showed that there were at least 11 recombinant clusters that have been formed with capability to transmit locally, in addition to the conventional subtype clusters. The newly formed clusters were considered as pCRFs to distinguish them with conventional CRFs that circulating in China, while other recombinants referred to URFs. The emergence of the pCRFs and URFs were uneven and statistically correlated with residency outside the prefecture capital, migration from Myanmar, and lower education levels.

Within pCRFs clusters, nearly all structures were composed of a subtype C genomic backbone, except for one whose genome consisted of roughly one third subtype B’, C, and 01_AE. Previous studies found that CRF01_AE strains were mainly associated with sexual transmission in China, while the B’/C recombinant subtypes were mainly associated with intravenous drug use^3^. Hence, it is likely that the pCRFs observed in the predominantly sexually transmitted populations in our study were related to HIV-1 strains originated from earlier IDU epidemics. Further studies are needed to clarify this hypothesis.

Dehong is China’s first HIV-1 epidemic region and past studies indicated that subtype B’, C, CRF07_BC and CRF08_BC were all introduced to China through Dehong^4^,^5^,^6^,^7^,^8^,^9^. In this study, eleven pCRFs are found, indicating that the recombinant strains are already obtaining the ability to spread in this region. It is reported that recombination is more evolutionarily advantageous than point mutations, since it introduces a large number of mutations while maintaining the capability of transmission^28^. In addition to the new recombinants’ transmission ability, other facilitating factors will further determine its transmission impact. The drug trafficking routes through Dehong facilitated the spread of the CRF07_BC and CRF08_BC from Thailand, Myanmar, and Laos to other areas of China in the 1990s. With great effort put on crackdown drug trafficking and methadone treatment to IDUs, the HIV incidence in IDUs of Dehong has declined^29^. However, the identification of multiple circulating recombinant HIV-1 strains in local sexually active youths is worrisome, considering the gateway position of Dehong in China’s HIV epidemic history. Meanwhile, recent promotion of border tourism and trades may introduce millions of people from other parts of China ( http://www.dehong.gov.cn/), increasing the transmission risk of novel recombinant HIV-1 strains. The situation therefore deserves attention from local and national public authorities.

In this study, there are more Myanmar that sampled in 2011 and 2012 than the two previews years. This may be related to concurrent conflicts in northern Myanmar, which increased the outgoing refugee count from northern Myanmar to Dehong ( http://www.unhcr.org/5049e42b6.html). For historical reasons, people in Dehong and Myanmar along the border share close relationships. Our study found that the newly formed URFs were significantly related to the Myanmar residents near the border, in which more than half are of Jingpo ethnicity, an ethnic minority group that inhabits both sides of the China-Myanmar border. In statistical analyses, high risk contact history showed no effect on URF generation, but a close association is observed between IDU status and Jingpo ethnicity (Supplemental Table S3, p = 0.005). In consideration of the fact of higher probability of recombination in IDUs, it is speculated that the Jingpo ethnicity in Myanmar may play an important role in the formation of recombinant strains. Meanwhile, significant association was also found between URFs generation and lower education levels, suggesting that those youths are most at risk for multiple infections and recombinant infections.

Since 1980s, the local governments have paid much effort on HIV prevention and control. Dehong is one of the few places in China where the number of reported HIV infection cases have experienced a plateau or slight decline in recent years^29^. However, the high proportion of new recombinant HIV-1 strains among recently infected youths, as well as increasing cross-border migration, indicate that the fight against AIDS in Dehong may still be far from over. The challenges for further controlling the HIV epidemic in Dehong include: 1) heterosexual and IDU transmissions are intertwined in overlapping populations, which facilitated the recombinant strain formation and transmission; 2) cross-border migrations of both Burmese and Chinese residents; 3) difficulties in reaching people with lower education levels and those living in remote regions beyond the prefecture capital. The findings of the study also indicated that monitoring the prevalence of HIV recombinants has important implications for assessing the overall risk exposure to HIV infection and for serving as an early warning indicator for potential outbreaks by new viral recombinants.

As the low hanging fruits in AIDS prevention had been picked up, redoubled efforts are needed to implement in-depth interventions and leave no untouched stones. More specific prevention measures should be targeted to reduce the multiple risk exposures in both the IDUs and heterosexuals populations in Dehong. Such efforts include continued crackdown on drug trafficking and improve harm reduction services, such as methadone treatment clinics, needle exchanges programs and condom promotion, to the last mile reaching people with low education and living in remote areas As the spread of infectious disease recognizes no border, so do the control efforts. The Dehong government should extend regular information sharing with the neighboring Burmese authorities to coordinate synergistic and joint HIV/AIDS control and intervention programs. The success of Dehong’s AIDS control campaign will rely on collaborative prevention and control efforts on both sides of the border.

The study has some limitations. Since the epidemic of HIV-1 in Dehong is quite unique, and many recombinant strains have been reported here, using of short gene segments (approximately 1200 bps) to define the genotype may underestimate the variety of and frequency of recombinants. We have attempted amplification of pol and gag fragments and near full-length genomes for as many samples as possible; however, they were not obtained for all strains due to limited blood plasma stock and long term cryopreservation.

## Methods

### Ethics Statement

The use of Human Blood Plasma in the current study has been approved by the institutional review boards of the National Center for AIDS/STD Control and Prevention, Chines Center for Disease Control and Prevention. All study participants gave their written informed consent. The methods were carried out in “accordance” with the approved guidelines. All experimental protocols were approved by the institutional review boards of the National Center for AIDS/STD Control and Prevention, China CDC.

### Study population

The study population was selected according to the WHO Threshold Survey method for transmitted HIV drug resistance surveillance in 2009 to 2012^30^. Per national regulations, newly diagnosed cases of HIV infection are reported to the Centers for Disease Control and Prevention (CDC) system at the county, prefecture, province, and national level within 24 hours ( http://english.gov.cn/laws/2005-10/10/content_75718.htm). All HIV-1 infected persons of less than 25 year old reported to the county CDCs in Dehong prefecture were consecutively enrolled. If less than 34 eligible participants were enrolled by the end of a three-month period, the sampling period was extended until the minimum sample size was reached. Participants were excluded if their CD4^+^ cell counts <500 cells/ml and, if female, had previous pregnancy histories. Basic demographic information, risk behavior histories, and a blood sample were acquired from each participant following written informed consent. Participants infected through mother-to-child transmission were further excluded from this study.

### Genotyping

Blood plasma samples were subjected to viral RNA extraction and cDNA synthesis as previously described^31^,^32^. A 1208 base-pair (bp) segment of the HIV-1 *pol* gene (HXB2 coordinates: 2242–3450 nt) was amplified and sequenced using an in-house drug resistance genotyping method^31^. Briefly, viral RNA was extracted from patient blood plasma using the QIAamp Viral RNA Mini Kit (Qiagen, Germany). cDNA transcription and first-round polymerase chain reaction (PCR) was performed by one-step RT-PCR using primers RT21 (CTGTATTTCAGCTATCAAGTCTTTTGATGGG) and MAW26 (TGGAAATGTGGAAAA GAAGGAC). The primers of second-round PCR were PRO-1 (CAGAGCCAACAGCCCCACCA) and RT4R (CTGCCAATTCTAATTCTGCTTC). PCR products were purified and sequenced with an ABI PRISM 3100 DNA sequencer (Applied Biosystems, USA). For further confirmation of the sequence subtype, a segment of *gag* gene (790–1862) was also amplified.

For near full-length genome (NFLG) amplification, RNA was reverse transcribed and cDNA with near-end point dilution was amplified followed by positive PCR products purifying and sequencing^32^. Phylogenetic analysis was used to identify the subtype of sequences with the standard reference sequences downloading form Los Alamos database. Phylogenetic analysis was performed using the maximum likelihood (ML) method in PhyML^33^. The general time reversible (GTR) plus gamma model of nucleotide substitution was selected by FindModel ( http://www.hiv.lanl.gov) to construct ML trees. Branch significance was analyzed by bootstrap with 200 replicates. Recombinants including CRF07_BC, CRF08_BC and sequences that cannot be classified into B, C and CRF01_AE were verified by bootscan analysis using SimPlot v 3.5.1 with window size of 120 bp and step size of 10 bp^34^ and Recombinant Identification Program (RIP)^35^.

### Statistical analysis

Samples were divided into three categories according to the genotypes of their recombinant forms: conventional subtypes, potential circulating recombinant forms (pCRFs), and URFs. The conventional subtype groups include subtypes B’, C, CRF01_AE, CRF07_BC, and CRF08_BC, which are widely prevalent in China^3^. The URFs group refers to newly formed or non-propagating recombinant strains, while the pCRFs group consists of strains with three or more samples clustered in phylogenetic clusters with high bootstrap values (≥0.7), indicating strains that are currently in local circulation with limited range of spread. The associations between demographic risk factors and infection by the three categories were analyzed using Chi-square tests and univariate and multivariate logistic regression with both the forward selection and backward elimination stepwise methods. In multivariate regression analysis, risk behavior history categories with very few cases such as men-who-have-sex-with-men and those with unknown routes of infection, were excluded from analysis. A two-sided p value of 0.05 or less was regarded as significant. All statistical analysis was performed using SPSS Statistics version 19.

## Additional Information

**How to cite this article**: Wei, H. *et al.* The sexually driven epidemic in youths in China's southwestern border region was caused by dynamic emerging multiple recombinant HIV-1 strains. *Sci. Rep.* **5**, 11323; doi: 10.1038/srep11323 (2015).

## Supplementary Material

Supplementary Information

## Figures and Tables

**Table 1:** Basic characteristics of study population.

	N	% Total
Total	205	100%
Year of diagnosis		
2009	54	26.3%
2010	49	23.9%
2011	43	21.0%
2012	59	28.8%
Sex		
Female	104	50.7%
Male	101	49.3%
Marital status		
Married or co-habiting	102	49.8%
Single, separated, or divorced	103	50.2%
Ethnicity		
Han	83	40.5%
Dai (minority)	47	22.9%
Jingpo (minority)	63	30.7%
Other (minorities)	12	5.9%
Education level		
No schooling	47	22.9%
Primary school	76	37.1%
Junior school	69	33.7%
High school and above	13	6.3%
Occupation		
Farmer	153	74.6%
Not farmer	52	25.4%
Residence		
Luxi County	83	40.5%
Other Dehong county	76	37.1%
Myanmar	36	17.6%
Other region in China	10	4.9%
High-risk contact history		
Heterosexual	152	74.1%
IDU	40	19.5%
MSM	6	2.9%
N/A	7	3.4%

Newly diagnosed HIV infection cases in young people ≤25 years old consecutively reported in approximately 3 months of sampling each year from 2009 to 2012.

**Table 2:** Multiple Logistic regression analysis of factors associated with infection with pCRFs and URFs, as compared to conventional subtypes (B, C, CRF01_AE, CRF07_BC, and CRF08_BC).

Total, n (%)	Conventional		pCRFs		URFs		pCRFs		URFs	
	72	(35.1%)	54	(26.3%)	79	(38.5%)	Crude OR (95% CI)	Adjusted OR (95% CI)	Crude OR (95% CI)	Adjusted OR (95% CI)
Year of diagnosis										
2009	22	(40.7%)	20	(37.0%)	12	(2.2%)	1		1	
2010	20	(40.8%)	12	(24.5%)	17	(34.7%)	0.66 (0.26–1.69)		1.56 (0.60–4.05)	
2011	12	(27.9%)	7	(16.3%)	24	(55.8%)	0.64 (0.21–1.95)		3.67 (1.37–9.84)*	
2012	18	(30.5%)	15	(25.4%)	26	(44.1%)	0.92 (0.37–2.29)		2.69 (1.05–6.68)*	
Sex										
Female	41	(39.4%)	28	(26.9%)	35	(33.7%)	1		1	
Male	31	(30.7%)	26	(25.7%)	44	(43.6%)	1.23 (0.60–2.50)		1.66 (0.87–3.17)	
Ethnicity										
Han	35	(42.2%)	24	(28.9%)	24	(28.9%)	1		1	
Jingpo (minority)	16	(25.4%)	19	(30.2%)	28	(44.4%)	1.73 (0.75–4.03)		2.55 (1.14–5.71)*	
Other minority	21	(35.6%)	11	(18.6%)	27	(45.8%)	0.76 (0.31–1.87)		1.87 (0.87–4.06)	
Education level										
Junior and above	36	(43.9%)	28	(34.1%)	18	(22.0%)	1	1	1	1
No schooling and Primary	36	(29.3%)	26	(21.1%)	61	(49.6%)	0.93 (0.46–1.88)	0.81 (0.38–1.74)	3.39 (1.68– 6.82)*	2.541(1.09–5.36)*
Occupation										
Not farmer	48	(31.4%)	40	(26.1%)	65	(42.5%)	1		1	
Farmer	24	(46.2%)	14	(26.9%)	14	(26.9%)	1.43 (0.65–3.12)		2.32 (1.09–4.95)*	
Residence										
Luxi County	37	(44.6%)	26	(31.3%)	20	(24.1%)	1	1	1	1
Other Dehong county	21	(27.6%)	23	(30.3%)	32	(42.1%)	1.56 (0.72–3.39)	1.56 (0.70–3.49)	2.82(1.30–6.11)*	2.90 (1.28–6.60)*
Myanmar	10	(27.8%)	2	(5.6%)	24	(66.7%)	0.29 (0.06–1.41)	0.28 (0.54–1.41)	4.44 (1.78–11.1) *	3.17 (1.20–8.39)*
Other region in China	4	(40.0%)	3	(30.0%)	3	(30.0%)	1.07 (0.22–5.18)	1.18 (0.21–6.51)	1.39 (0.28–6.82)	1.79 (0.26–12.4)
High-risk contact history										
Heterosexual	54	(35.5%)	43	(28.3%)	55	(36.2%)	1		1	
Injecting drug use	10	(25.0%)	10	(25.0%)	20	(50.0%)	1.26 (0.48–3.29)		1.96 (0.84–4.58)	
MSM€	4	(66.7%)	0	(0%)	2	(33.3%)	–		–	
Not available€	4	(57.1%)	2	(28.6%)	1	(14.3%)	–		–	

^*^OR greater than one for both upper and lower bound of 95% CI.

^€^The high-risk contact category “MSM” , as well as the “Not available” category was excluded from analysis due to its small number of cases. Cases were designated missing “-”.
