# Supplementary material for: The sexually driven epidemic in youths in China’s southwestern border region was caused by dynamic emerging multiple recombinant HIV-1 strains
Source: Sci Rep. 2015 Jul 2;5:11323. doi: 10.1038/srep11323 (PMC4489098; doi:10.1038/srep11323)
Supplement: Supplementary Information [file srep11323-s1.pdf]

The sexually driven epidemic in youths in China's southwestern border region was caused by dynamic emerging multiple recombinant HIV-1 strains

Huamian Wei[1]*, Hui Xing[1]*, Jenny H. Hsi[1]*, Manhong Jia[2]*, Yi Feng[1], Song Duan[3], Cui He[1], Shitang Yao[3], Yuhua Ruan[1], Xiang He[4], Lingjie Liao[1], Yanling Ma[2], Yunda Huang[5], Lin Lu[2], Yiming Shao[1 #]

1. State Key Laboratory for Infectious Disease Prevention and Control, Collaborative Innovation Center for Diagnosis and Treatment of Infectious Diseases, National Center for AIDS/STD Control and Prevention, Chinese Center for Disease Control and Prevention, Beijing, China
2. Yunnan Center for Disease Control and Prevention, Kunming, Yunnan, China
3. Dehong Center for Disease Control and Prevention, Dehong, Yunnan, China
4. Guangdong Provincial Institute of Public Health, Guangdong, China
5. Fred Hutchinson Cancer Research Center, Seattle, USA

[*] Joint first authors

[#] Correspondence should be addressed to:

Yiming Shao, PhD, MD

State Key Laboratory for Infectious Disease Prevention and Control and National Center for AIDS/STD Control and Prevention, Chinese Center for Disease Control and Prevention,

155 Changbai Road Changping District, Beijing 102206, China.

Email: yshao08@gmail.com

Supplemental Table S1. The subtype list of each sequence. The segment of *pol* (HXB2:2242-3450), *gag* (790-1862) and near full-length genome was amplified. The subtype was identified by phylogenetic trees, Recombinant Identification Program (RIP) and Simplot software.

**Patient ID**	**Subtype**			**Integrated Subtype***
	pol	***gag***	**NFLG**	
DH09JJX01	C	N/A	N/A	BC_pCRF2
DH09JJX02	BC	N/A	N/A	01BC_pCRF7
DH09JJX03	B	N/A	N/A	B
DH09JJX04	BC	N/A	N/A	BC_pCRF11
DH09JJX05	CRF01_AE	N/A	N/A	CRF01_AE
DH09JJX06	BC	N/A	N/A	BC
DH09JJX07	BC	N/A	N/A	BC_pCRF3
DH09JJX08	BC	N/A	N/A	BC
DH09JJX09	BC	BC	N/A	BC
DH09JJX10	CRF08_BC	N/A	N/A	CRF08_BC
DH09JJX11	BC	N/A	N/A	BC
DH09JJX12	C	C	N/A	C
DH09JJX13	BC	N/A	N/A	BC_pCRF3
DH09JJX14	C	01BC	N/A	01BC
DH09JJX15	01BC	C	N/A	01BC
DH09JJX16	BC	N/A	N/A	BC
DH09JJX17	C	N/A	N/A	BC_pCRF5
DH09JJX18	C	N/A	N/A	BC_pCRF2
DH09JJX19	C	N/A	N/A	01BC_pCRF4
DH09JJX20	C	01BC	01BC	01BC_pCRF4
DH09JJX21	BC	BC	BC	BC
DH09JJX22	C	C	C	C
DH09JJX23	C	C	N/A	C
DH09JJX24	BC	BC	N/A	BC_pCRF3
DH09JJX25	C	BC	N/A	C
DH09JJX26	B	N/A	N/A	B
DH09JJX27	B	N/A	N/A	B
DH09JJX28	CRF01_AE	N/A	N/A	CRF01_AE
DH09JJX29	CRF01_AE	N/A	N/A	CRF01_AE
DH09JJX30	BC	N/A	N/A	01BC_pCRF10
DH09JJX31	C	C	BC	BC_pCRF5
DH09JJX32	BC	AE	N/A	01BC
DH09JJX33	CRF01_AE	N/A	N/A	CRF01_AE
DH09JJX34	01B	N/A	N/A	01B
DH09JJX35	C	BC	N/A	01BC_pCRF4
DH09JJX36	BC	BC	N/A	BC
DH09JJX37	CRF01_AE	N/A	N/A	CRF01_AE
DH09JJX38	CRF08_BC	N/A	N/A	CRF08_BC

Patient ID	Subtype			Integrated Subtype*
	pol	*gag*	NFLG	
DH09JJX39	C	BC	N/A	C
DH09JJX40	BC	N/A	N/A	BC_pCRF3
DH09JJX41	C	C	C	C
DH09JJX42	BC	BC	N/A	BC_pCRF3
DH09JJX43	BC	N/A	N/A	BC_pCRF3
DH09JJX44	B	N/A	N/A	B
DH09JJX45	C	N/A	N/A	C
DH09JJX46	BC	BC	BC	BC_pCRF3
DH09JJX47	CRF07_BC	N/A	N/A	CRF07_BC
DH09JJX48	CRF01_AE	N/A	N/A	CRF01_AE
DH09JJX49	BC	N/A	N/A	BC
DH09JJX50	C	01BC	N/A	01BC_pCRF4
DH09JJX51	C	N/A	N/A	C
DH09JJX52	CRF08_BC	N/A	N/A	CRF08_BC
DH09JJX53	BC	N/A	N/A	BC_pCRF8
DH09JJX54	BC	N/A	N/A	BC_pCRF3
DH10JJX01	BC	N/A	N/A	01BC_pCRF1
DH10JJX02	CRF01_AE	AE	AE	CRF01_AE
DH10JJX03	CRF01_AE	N/A	N/A	CRF01_AE
DH10JJX04	BC	BC	N/A	BC
DH10JJX05	CRF08_BC	CRF08_BC	N/A	CRF08_BC
DH10JJX06	CRF01_AE	N/A	N/A	CRF01_AE
DH10JJX07	BC	BC	N/A	BC
DH10JJX08	BC	01C	01BC	01BC_pCRF10
DH10JJX09	BC	C	BC	BC
DH10JJX10	01C	N/A	N/A	01C
DH10JJX11	C	C	N/A	C
DH10JJX12	BC	AE	N/A	01BC
DH10JJX13	CRF01_AE	N/A	N/A	CRF01_AE
DH10JJX14	BC	N/A	N/A	BC_pCRF3
DH10JJX15	CRF01_AE	N/A	N/A	CRF01_AE
DH10JJX16	C	N/A	N/A	C
DH10JJX17	CRF08_BC	N/A	N/A	CRF08_BC
DH10JJX18	C	01BC	01BC	01BC_pCRF4
DH10JJX19	BC	N/A	N/A	BC
DH10JJX20	BC	N/A	N/A	BC
DH10JJX21	C	N/A	N/A	C
DH10JJX22	CRF08_BC	N/A	N/A	CRF08_BC
DH10JJX23	CRF01_AE	N/A	N/A	CRF01_AE
DH10JJX24	BC	N/A	N/A	BC_pCRF3
DH10JJX25	01BC	BC	01BC	01BC
DH10JJX26	BC	BC	BC	BC_pCRF3

Patient ID	Subtype			Integrated Subtype*
	pol	*gag*	NFLG	
DH10JJX27	CRF01_AE	AE	AE	CRF01_AE
DH10JJX28	C	N/A	N/A	C
DH10JJX29	C	01BC	01BC	01BC_pCRF4
DH10JJX30	CRF08_BC	N/A	N/A	CRF08_BC
DH10JJX31	BC	C	N/A	BC
DH10JJX32	BC	BC	N/A	01BC_pCRF1
DH10JJX33	B	N/A	N/A	B
DH10JJX34	C	BC	BC	BC_pCRF2
DH10JJX35	01C	AE	01C	01C
DH10JJX36	01C	N/A	N/A	01C
DH10JJX37	CRF01_AE	N/A	N/A	CRF01_AE
DH10JJX38	BC	BC	N/A	BC
DH10JJX39	BC	BC	N/A	BC
DH10JJX40	BC	BC	N/A	BC
DH10JJX41	BC	N/A	N/A	BC
DH10JJX42	01C	AE	N/A	01C
DH10JJX43	BC	C	01BC	01BC_pCRF7
DH10JJX44	BC	BC	BC	BC
DH10JJX45	BC	C	BC	BC_pCRF11
DH10JJX46	C	N/A	N/A	C
DH10JJX47	CRF07_BC	N/A	N/A	CRF07_BC
DH10JJX48	BC	N/A	N/A	BC_pCRF9
DH10JJX49	C	N/A	N/A	C
DH11JJX01	B	N/A	N/A	B
DH11JJX02	C	N/A	N/A	BC_pCRF5
DH11JJX03	BC	N/A	N/A	BC
DH11JJX04	C	N/A	N/A	01BC_pCRF6
DH11JJX05	01BC	N/A	N/A	01BC
DH11JJX06	BC	01BC	N/A	01BC
DH11JJX07	BC	C	N/A	BC
DH11JJX08	CRF01_AE	N/A	N/A	CRF01_AE
DH11JJX09	BC	N/A	N/A	BC
DH11JJX10	BC	N/A	N/A	BC
DH11JJX11	B	N/A	N/A	B
DH11JJX12	C	N/A	N/A	C
DH11JJX13	BC	N/A	N/A	BC
DH11JJX14	BC	AE	N/A	01BC
DH11JJX15	C	N/A	N/A	C
DH11JJX16	CRF08_BC	N/A	N/A	CRF08_BC
DH11JJX17	CRF01_AE	N/A	N/A	CRF01_AE
DH11JJX18	BC	N/A	N/A	01BC_pCRF10
DH11JJX19	CRF07_BC	N/A	N/A	CRF07_BC

Patient ID	Subtype			Integrated Subtype*
	pol	***gag***	**NFLG**	
DH11JJX20	01C	01BC	N/A	01BC
DH11JJX21	01C	N/A	N/A	01C
DH11JJX22	B	N/A	N/A	B
DH11JJX23	BC	N/A	N/A	BC_pCRF11
DH11JJX24	BC	N/A	N/A	BC
DH11JJX25	BC	N/A	N/A	BC
DH11JJX26	BC	N/A	N/A	BC
DH11JJX27	C	N/A	N/A	C
DH11JJX28	BC	N/A	N/A	BC
DH11JJX29	C	N/A	N/A	C
DH11JJX30	01BC	N/A	N/A	01BC
DH11JJX31	01BC	N/A	N/A	01BC
DH11JJX32	01C	N/A	N/A	01C
DH11JJX33	C	N/A	N/A	01BC_pCRF4
DH11JJX34	BC	N/A	N/A	BC
DH11JJX35	BC	N/A	N/A	BC
DH11JJX36	BC	N/A	N/A	BC
DH11JJX37	BC	N/A	N/A	BC
DH11JJX38	BC	N/A	N/A	BC
DH11JJX39	BC	N/A	N/A	01BC_pCRF7
DH11JJX40	01C	N/A	N/A	01C
DH11JJX41	BC	N/A	N/A	BC
DH11JJX42	BC	N/A	N/A	01BC_pCRF10
DH11JJX43	CRF01_AE	N/A	N/A	CRF01_AE
DH12JJX01	01C	N/A	N/A	01C
DH12JJX02	CRF01_AE	N/A	N/A	CRF01_AE
DH12JJX03	BC	BC	BC	BC
DH12JJX04	01BC	N/A	N/A	01BC
DH12JJX05	BC	BC	BC	BC_pCRF8
DH12JJX06	CRF01_AE	AE	N/A	CRF01_AE
DH12JJX07	CRF01_AE	AE	N/A	CRF01_AE
DH12JJX08	CRF01_AE	N/A	N/A	CRF01_AE
DH12JJX09	BC	N/A	N/A	BC_pCRF9
DH12JJX10	BC	N/A	N/A	BC
DH12JJX11	BC	BC	N/A	BC
DH12JJX12	BC	N/A	N/A	01BC_pCRF10
DH12JJX13	CRF08_BC	N/A	N/A	CRF08_BC
DH12JJX14	BC	N/A	N/A	BC_pCRF3
DH12JJX15	CRF08_BC	N/A	N/A	CRF08_BC
DH12JJX16	CRF01_AE	N/A	N/A	CRF01_AE
DH12JJX17	CRF01_AE	AE	N/A	CRF01_AE
DH12JJX18	BC	BC	N/A	BC_pCRF3

Patient ID	Subtype			Integrated Subtype*
	pol	***gag***	**NFLG**	
DH12JJX19	01C	N/A	N/A	01C
DH12JJX20	BC	N/A	N/A	BC
DH12JJX21	CRF01_AE	AE	N/A	CRF01_AE
DH12JJX22	BC	01BC	N/A	01BC
DH12JJX23	C	BC	01BC	01BC_pCRF6
DH12JJX24	C	01BC	N/A	01BC_pCRF4
DH12JJX25	BC	N/A	N/A	BC
DH12JJX26	BC	N/A	N/A	BC
DH12JJX27	C	N/A	N/A	BC_pCRF2
DH12JJX28	BC	N/A	N/A	BC
DH12JJX29	C	C	N/A	C
DH12JJX30	CRF01_AE	N/A	N/A	CRF01_AE
DH12JJX31	BC	N/A	N/A	BC
DH12JJX32	CRF01_AE	AE	N/A	CRF01_AE
DH12JJX33	01BC	N/A	N/A	01BC
DH12JJX34	BC	BC	N/A	BC
DH12JJX35	BC	N/A	N/A	BC
DH12JJX36	BC	N/A	N/A	BC
DH12JJX37	BC	N/A	N/A	BC
DH12JJX38	C	N/A	N/A	01BC_pCRF6
DH12JJX39	C	N/A	N/A	C
DH12JJX40	BC	N/A	N/A	BC
DH12JJX41	C	BC	N/A	BC_pCRF2
DH12JJX42	C	01B	N/A	01BC_pCRF4
DH12JJX43	BC	C	01BC	01BC_pCRF1
DH12JJX44	BC	AE	N/A	01BC
DH12JJX45	CRF01_AE	BC	N/A	01BC
DH12JJX46	CRF01_AE	N/A	N/A	CRF01_AE
DH12JJX47	BC	BC	BC	BC_pCRF9
DH12JJX48	CRF01_AE	AE	N/A	CRF01_AE
DH12JJX49	01B	01BC	N/A	01BC
DH12JJX50	BC	N/A	N/A	BC_pCRF3
DH12JJX51	BC	N/A	N/A	BC
DH12JJX52	01C	N/A	N/A	01C
DH12JJX53	C	N/A	N/A	C
DH12JJX54	CRF01_AE	N/A	N/A	CRF01_AE
DH12JJX55	BC	N/A	N/A	BC
DH12JJX56	BC	BC	N/A	BC_pCRF8
DH12JJX57	BC	BC	BC	BC
DH12JJX58	B	B	N/A	B
DH12JJX59	BC	N/A	N/A	BC

N/A: not available.

* The subtype of each sequence was finally fixed according to the results of *pol*, *gag* and NFLG. The subtype of sequences in each clade, such as pCRFs, referred to the subtype of NFLG sequences in their respective clades.

Supplemental Table S2 Chi-square analysis of factors associated with different subtype categories. The case count (n) for each category is listed, along with its percentage within that demographic group (row percentage, %). Conventional_AE denotes CRF01_AE. Conventional _BC denotes B'/C subtype (B', C, CRF07_BC, and CRF08_BC). pCRFs_AE, pCRFs_BC, URFs_AE, and URFs_BC denote the recombinant categories that do and do not include CRF01_AE fragments, respectively.

	Conventional _AE		Conventional _BC		pCRFs_AE		pCRFs_BC		URFs_AE		URFs_BC		P value
Total, n (%)	29	(14.1 %)	43	(21.0%)	23	(11.2%)	31	(15.1%)	28	(13.7%)	51	(24.9%)	
Year of diagnosis													0.055
2009	6	(11.1%)	16	(29.6%)	6	(11.1%)	14	(25.9%)	4	(7.4%)	8	(14.8%)	
2010	8	(16.3%)	12	(24.5%)	6	(12.2%)	6	(12.2%)	6	(12.2%)	11	(22.4%)	
2011	3	(7.0%)	9	(20.9%)	5	(11.6%)	2	(4.7%)	9	(20.9%)	15	(34.9%)	
2012	12	(20.3%)	6	(10.2%)	6	(10.2%)	9	(15.3%)	9	(15.3%)	17	(28.8%)	
Sex													0.154
Female	15	(14.4%)	26	(25.0%)	16	(15.4%)	12	(11.5%)	12	(11.5%)	23	(22.1%)	
Male	14	(13.9%)	17	(16.8%)	7	(6.9%)	19	(18.8%)	16	(15.8%)	28	(27.7%)	
Marital status													0.331
Married	11	(10.8%)	18	(17.6%)	12	(11.8%)	19	(18.6%)	17	(16.7%)	25	(24.5%)	
Single	18	(17.5%)	25	(24.3%)	11	(10.7%)	12	(11.7%)	11	(10.7%)	26	(25.2%)	
Ethnicity													0.001
Han	21	(25.3%)	14	(16.9%)	14	(16.9%)	10	(12.0%)	11	(13.3%)	13	(15.7%)	
Jingpo	1	(1.6%)	15	(23.8%)	3	(4.8%)	16	(25.4%)	7	(11.1%)	21	(33.3%)	
Dai	7	(14.9%)	13	(27.7%)	4	(8.5%)	4	(8.5%)	8	(17.0%)	11	(23.4%)	
Other minorities	0	-	1	(8.3%)	2	(16.7%)	1	(8.3%)	2	(16.7%)	6	(50.0%)	
Education level													< 0.001
No schooling	4	(8.5%)	6	(12.8%)	2	(4.3%)	5	(10.6%)	5	(10.6%)	25	(53.2%)	
Primary school	8	(10.5%)	18	(23.7%)	4	(5.3%)	15	(19.7%)	16	(21.1%)	15	(19.7%)	
Junior school	12	(17.4%)	15	(21.7%)	14	(20.3%)	11	(15.9%)	7	(10.1%)	10	(14.5%)	
High school and above	5	(38.5%)	4	(30.8%)	3	(23.1%)	0	-	0	-	1	(7.7%)	
Occupation													0.012
Farmer	15	(9.8%)	33	(21.6%)	14	(9.2%)	26	(17.0%)	24	(15.7%)	41	(26.8%)	
Not farmer	14	(26.9%)	10	(19.2%)	9	(17.3%)	5	(9.6%)	4	(7.7%)	10	(19.2%)	

	Conventional_AE		Conventional_BC		pCRFs_AE		pCRFs_BC		URFs_AE		URFs_BC		P value
Residence													0.003
Luxi County	14	(16.9%)	23	(27.7%)	11	(13.3%)	15	(18.1%)	10	(12.0%)	10	(12.0%)	
Other Dehong county	7	(9.2%)	14	(18.4%)	10	(13.2%)	13	(17.1%)	9	(11.8%)	23	(30.3%)	
Myanmar	5	(13.9%)	5	(13.9%)	1	(2.8%)	1	(2.8)	6	(16.7%)	18	(50.0%)	
Other region in China	3	(30.0%)	1	(10.0%)	1	(10.0%)	2	(20.0%)	3	(30.0%)	0	-	
High-risk contact history													0.002*
Heterosexual	22	(15.1%)	32	(21.7%)	22	(14.5%)	21	(13.8%)	21	(8.6%)	34	(26.3%)	
Injecting drug use	1	(2.5%)	9	(22.5%)	1	(2.5%)	9	(22.5%)	5	(12.5%)	15	(37.5%)	
MSM	4	(66.7%)	0	-	0	-	0	-	1	(16.7%)	1	(16.7%)	
Not available	2	(28.6%)	2	(28.6%)	0	-	1	(14.3%)	1	(14.3%)	1	(14.3%)	

*people without clear high risk contact history was exclude from the dataset when doing χ -square analysis.

Supplemental table S3 Chi-square analysis of factors associated with different subtype categories. The case count (n) for each category is listed, along with its percentage within that demographic group (row percentage, %). “Conventional subtypes” includes subtypes B’ and C and CRF01_AE, CRF07_BC, and CRF08_BC.

	Conventional subtypes	pCRFs	URFs	P-value
Total, n (%)	72 (35.1%)	54 (26.3%)	79 (38.5%)	
Year of diagnosis				0.033
2009	22 (40.7%)	20 (37.0%)	12 (2.2%)	
2010	20 (40.8%)	12 (24.5%)	17 (34.7%)	
2011	12 (27.9%)	7 (16.3%)	24 (55.8%)	
2012	18 (30.5%)	15 (25.4%)	26 (44.1%)	
Sex				0.295
Female	41 (39.4%)	28 (26.9%)	35 (33.7%)	
Male	31 (30.7%)	26 (25.7%)	44 (43.6%)	
Marital status				0.121
Married	29 (28.9%)	31 (30.4%)	42 (41.2%)	
Single	43 (41.7%)	23 (22.3%)	37 (35.9%)	
Ethnicity				0.039
Han	35 (42.2%)	24 (28.9%)	24 (28.9%)	
Jingpo (minority)	16 (25.4%)	19 (30.2%)	28 (44.4%)	
Dai (minority)	20 (42.6%)	8 (17.0%)	19 (40.4%)	
Other (minorities)	1 (8.3%)	3 (25.0%)	8 (66.7%)	
Education level				< 0.001
No schooling	10 (21.3%)	7 (14.9%)	30 (63.8%)	
Primary school	26 (34.2%)	19 (25.0%)	31 (40.8%)	
Junior high school	27 (39.1%)	25 (36.2%)	17 (24.6%)	
High school and above	9 (69.2%)	3 (23.1%)	1 (7.7%)	
Occupation				0.088
Farmer	48 (31.4%)	40 (26.1%)	65 (42.5%)	
Not farmer	24 (46.2%)	14 (26.9%)	14 (26.9%)	
Residence				0.001
Luxi County	37 (44.6%)	26 (31.3%)	20 (24.1%)	
Other Dehong county	21 (27.6%)	23 (30.3%)	32 (42.1%)	
Myanmar	10 (27.8%)	2 (5.6%)	24 (66.7%)	
Other region in China	4 (40.0%)	3 (30.0%)	3 (30.0%)	
High-risk contact history				0.178*
Heterosexual	54 (35.5%)	43 (28.3%)	55 (36.2%)	
Injecting drug use	10 (25.0%)	10 (25.0%)	20 (50.0%)	
MSM	4 (66.7%)	0 (0%)	2 (33.3%)	
Not available	4 (57.1%)	2 (28.6%)	1 (14.3%)	

*people without clear high risk contact history was exclude from the dataset when doing χ -square analysis.

Supplemental Table S4 Multiple Logistic regression analysis of factors associated with IDU population, as compared to heterosexual group. The MSM population was excluded from analysis due to its small number of cases (n=6). P value refers to the differences between IDU and heterosexual population within each factor. P value of 0.05 or less was regarded as significant.

	IDU			
	Univariate Regression Crude OR (95% CI)	P value	Multivariate Regression Adjusted OR (95% CI)	P value
Year of diagnosis		<0.001		0.014
2009	1		**1**	
2010	0.22 (0.05 - 1.09)		**0.17 (0.03 - 1.06)**	
2011	1.46 (0.53 - 4.01)		**0.17 (0.02 - 1.06)**	
2012	2.46 (0.99 - 6.12)		**1.96 (0.45 -8 .52)**	
Sex		<0.001		<0.001
Female	1		**1**	
Male	33.51 (7.78 - 144.3)*		**27.25 (5.77 - 128.6)***	
Marital status		<0.001		0.04
Married	1		**1**	
Single	3.44 (1.56 - 7.38)*		**3.05 (1.02 - 9.14)***	
Ethnicity		<0.001		0.005
Han	1		**1**	
Jingpo	4.20 (1.76 - 10.03)*		**8.65 (2.21 - 33.12)***	
Other minority	1.46 (0.54 - 3.95)		**1.89 (0.50 - 7.16)**	
Education level		<0.001		
Junior and above	1			
Primary and lower	4.35 (1.72 - 10.97)*			
Occupation		<0.001		
Farmer	1			
Not farmer	0.41 (0.15 - 1.13)			
Residence		<0.001		0.001
Luxi County	1		**1**	
Other Dehong county	2.27 (0.86 - 5.99)		**1.68 (0.45 - 6.30)**	
Myanmar	10.29 (3.70 - 28.6)*		**12.79 (2.04 - 80.12)***	
Other region in China	1.39 (0.15 - 12.97)		**2.54 (0.15 - 43.14)**	

* OR greater than one for both upper and lower bound of 95% CI.

Supplemental Figure Legends

Supplemental Figure S1. Bootscanning analysis of recombination in the HIV-1 *pol* gene region. Boostcanning was performed using Simplot with windows size of 120 and step size of 10. The parental reference sequences used were: subtype B, RL 42 (blue); subtype C, 95IN21068 (orange); and CRF01_AE, CM240 (olive). All sample sequences (n=205) were subjected to analysis, but only those identified with recombinant breakpoints are shown here.

Supplemental Figure S2. Phylogenetic analysis on regional transmission. The 1.2 kb *pol* gene sequences of all known recombinant forms from China, India, Myanmar, Thailand, and Vietnam in the Los Alamos HIV Database were downloaded and compared to our sample sequences using neighbor-joining phylogeny with 1000 bootstrap replications. Colors denote sequences from different regions and sources. All clusters from the main phylogenetic tree containing pCRF sequences in this study were extracted and detailed displayed.

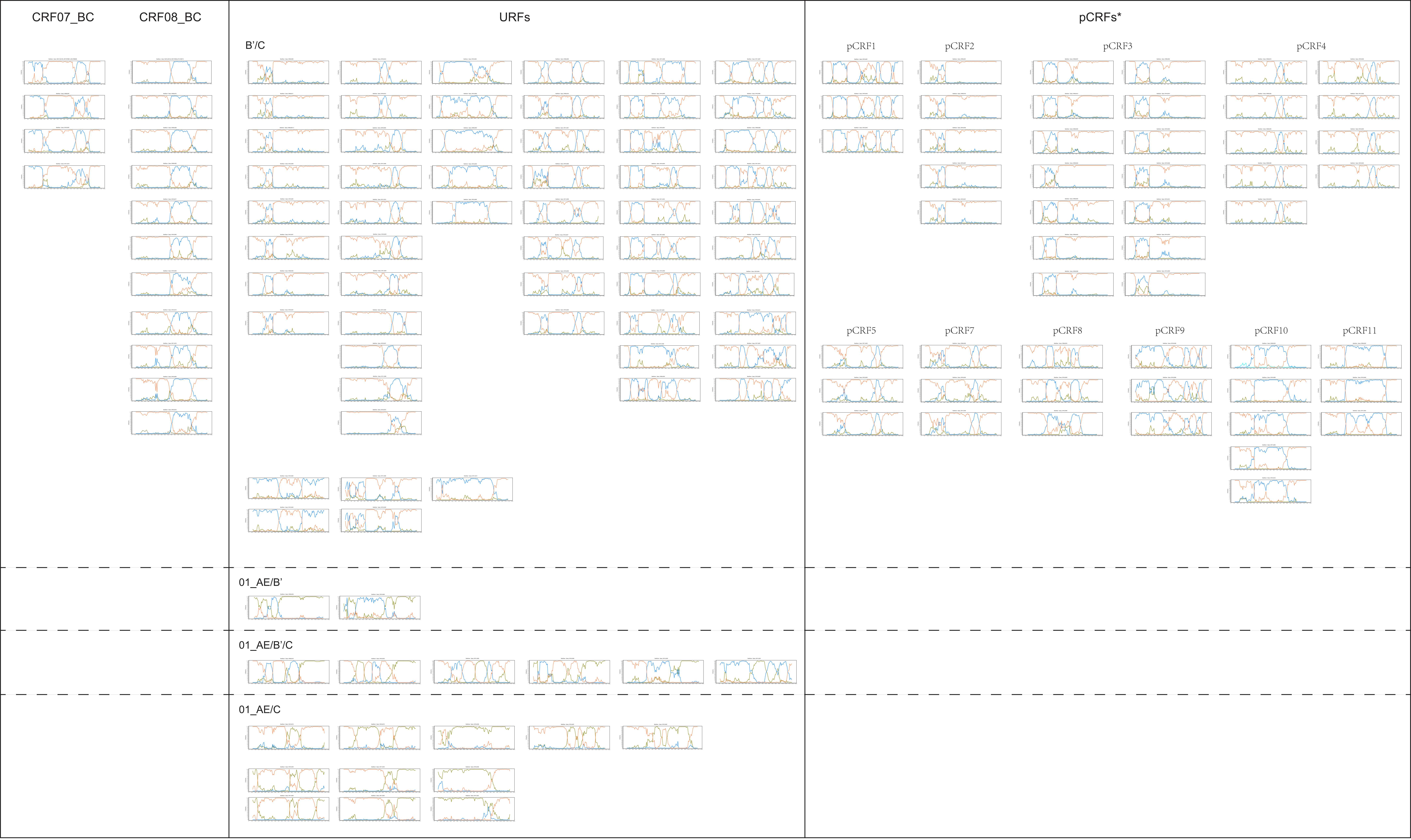

*pCRF6 was not shown because there was no recombinant in the correspondent region,

Figure S1

Figure S2

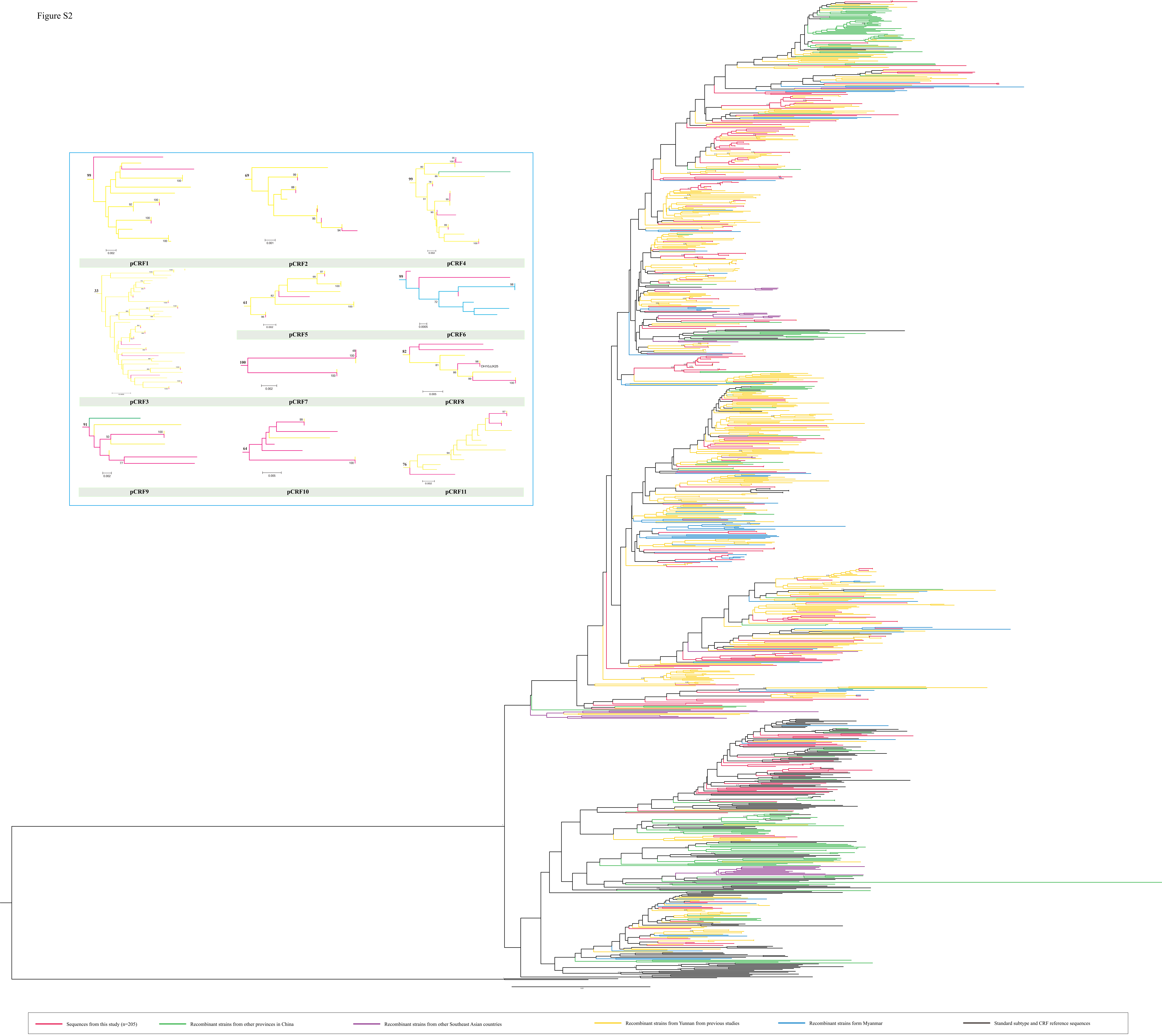
pCRF1
pCRF2
pCRF4
pCRF3
pCRF5
pCRF6
pCRF7
pCRF8
DH10JJX25
pCRF9
pCRF10
pCRF11
Sequences from this study (n=205)
Recombinant strains from other provinces in China
Recombinant strains from other Southeast Asian countries
Recombinant strains from Yunnan from previous studies
Recombinant strains form Myanmar
Standard subtype and CRF reference sequences
